# Cryo‐EM structures of amyloid β filaments from cotton wool plaques in Alzheimer disease caused by the PSEN1 V261I and A431E mutations

**DOI:** 10.1002/alz70855_098033

**Published:** 2025-12-23

**Authors:** Anllely Fernandez, Md Rejaul Hoq, Frank S. Vago, Grace Hallinan, Sakshibeedu R. Bharath, Daoyi Li, Kadir A. Ozcan, Holly Garringer, Wen Jiang, Ruben Vidal, Bernardino Ghetti

**Affiliations:** ^1^ Indiana University, INDIANAPOLIS, IN, USA; ^2^ Purdue University, West Lafayette, IN, USA

## Abstract

**Background:**

One of the neuropathologic phenotypes of Alzheimer disease (AD), associated with missense and deletion mutations in *PSEN1, is the presence of* cotton wool plaques (CWPs). CWPs are round amyloid β (Aβ) immunopositive structures, lacking an amyloid core; they are well recognizable in hematoxilin ‐eosin stained preparations and are not fluorescent using Thioflavin S (ThS), thus being morphologically different from Aβ cored plaques and Aβ diffuse deposits. Amino‐terminally truncated and post‐translationally modified Aβ peptide species are the main component of CWPs. Tau immunopositive neurites may be seen in CWPs and neurofibrillary tangles may be numerous amidst CWPs

**Method:**

We used cryo‐electron microscopy to study the structure of Aβ filaments extracted from the brains of two individuals affected by dominantly inherited AD caused by the *PSEN1* V261I and A431E mutations, respectively.

**Result:**

CWPs consist predominantly of two novel arrangements of type I Aβ filaments, and we have designated them as type Ic and type Id Aβ filaments. The latter filaments exhibit unique protofilament packing arrangements. While the type Ic Aβ filaments share a largely similar S‐shaped monomeric configuration with type I Aβ filaments, type Id Aβ filaments display an antiparallel and asymmetric packing, in contrast to the twofold parallel arrangement characteristic of type Ib and Ic Aβ filaments.

**Conclusion:**

The formation of type Ic and type Id Aβ filaments in CWPs may be linked to a distinct array of Aβ peptide species present in CWPs, differing from those found in core plaques in sporadic AD. This work provides novel insights into the role that different types of Aβ filaments may play in the composition of plaque morphology. Aggregation of Aβ as type Ic and type Id Aβ filaments may be the basis for the phenotype of CWPs. The characterization of Aβ filaments from CWPs, is important for the understanding of the role that the structure and assembly of amyloid peptides may play in the morphological diversity of Aβdeposits as well as for developing new diagnostic and therapeutic strategies for AD. (Supported by NIH P30‐AG010133, RF1‐NS110437and RF1‐AG071177)

